# Impact of a heart failure multidisciplinary clinic on the reduction of healthcare-related events and costs: the GEstIC study

**DOI:** 10.3389/fcvm.2023.1232291

**Published:** 2023-09-29

**Authors:** Rita Rego, Nuno Pereira, António Pinto, Sofia Pereira, Irene Marques

**Affiliations:** ^1^ Serviço de Medicina Interna, Centro Hospitalar Universitário de Santo António, Porto, Portugal; ^2^ Unidade Multidisciplinar de Investigação Biomédica—Instituto de Ciências Biomédicas de Abel Salazar (ICBAS), Universidade do Porto, Porto, Portugal; ^3^ Serviço de Medicina Interna, Centro Hospitalar de Tondela Viseu, Viseu, Portugal; ^4^ ITR-Laboratory for Integrative and Translational Research in Population Health, Porto, Portugal

**Keywords:** heart failure events, multidisciplinary management, heart failure clinic, hospitalization, urgent heart failure visit, healthcare utilization

## Abstract

**Introduction:**

Heart failure (HF) is the leading cause of hospitalization in the elderly in developed countries and significantly impacts public health expenditures. Patients with HF usually have associated comorbidities that require multidisciplinary management. This study aims to demonstrate the benefits of a multidisciplinary clinic in reducing all-cause hospitalizations and HF events (HF hospitalizations and urgent HF visits) in a real-world setting. Finally, the study evaluates the associated costs of HF events.

**Methods:**

This observational study included patients admitted to GEstIC, a multidisciplinary Portuguese HF clinic, from January 2013 to February 2019, who had one-year follow-up. Hospitalizations and HF events, total days spent in the hospital during HF hospitalizations, and HF events-related costs, in the year before and the year after GEstIC admission, were compared.

**Results:**

Of the 487 patients admitted to the GEstIC, 287 were eligible for the study sample. After one year of HF patients' multidisciplinary management at GEstIC, there was a 53.7% reduction in all-cause hospitalizations (462 vs. 214), a 71.7% reduction in HF hospitalizations (392 vs. 111), and a 39.1% reduction in urgent HF visits (87 vs. 53). As a result, there was a significant decrease of 12.6 days in the length of hospital stay due to HF per patient (15.6 vs. 3.0, *p* < 0.001). This translated into the release of 9.9 hospital beds in the year following admission to GEstIC. The average total savings associated with the reduction of HF events was €5,439.77 per patient (6,774.15 vs. 1,334.38, *p* < 0.001), representing a total cost reduction of €1,561,213. Furthermore, the significant reduction in the number of all events was independent of the patient's left ventricular ejection fraction (LVEF).

**Discussion:**

Significant reductions in all-cause and HF hospitalizations and urgent HF visits were observed with the implementation of this multidisciplinary clinic for HF patients' management. This was particularly important for patients with LVEF >40%. Before GEstIC, there was no medical intervention to improve the prognosis of these patients. The reduction of over one million euros in health-related costs after only one year of person-centered multidisciplinary management highlights the need to replicate this approach in other national healthcare institutions.

## 1. Introduction

Heart failure (HF) is the cardiovascular pandemic, affecting more than 60 million people worldwide, and is associated with significant morbidity and mortality (1). It is currently the leading cause of hospitalization in the elderly (>65 years) and a major cause of emergency hospital admissions (2, 3). In Portugal, there were 18,752 hospitalizations and 2,327 deaths for HF in 2016 (4). The in-hospital mortality rate, in 2014, was 12.5% (5). By 2035, the number of HF patients is expected to increase by 30%, mainly due to the aging of the population (6). Globally, HF represents a total economic burden of approximately $108 billion per year (7, 8). In Portugal, the total cost associated with HF reached €405 million in 2014, accounting for an economic burden of 2.6% of the public health expenditure (9).

Clinically, HF is often connected with a group of comorbidities, such as hypertension, diabetes, chronic kidney disease (CKD), atrial fibrillation, chronic obstructive pulmonary disease (COPD), cognitive dysfunction, anemia, and iron deficiency (10, 11). These conditions and their associated treatments may not only contribute to the progression of HF but also influence patients' response to treatment, thereby affecting their prognosis and quality of life (12). Thus, management and improvement of the associated comorbidities are crucial.

To address the complexity of HF patients’ management, most guidelines from cardiology societies advocate that patient care should be provided in a multidisciplinary manner, namely by establishing multidisciplinary clinics (2, 3, 13, 14). These clinics should be staffed by specialist physicians, nurses, pharmacists, nutritionists, psychologists, physiotherapists, and social workers, among others, so that they can guarantee adequate treatment and psychological support to patients while educating them about their disease (2, 3, 15–17). Managing HF patients through this multidisciplinary approach has been shown to reduce the mortality and number of HF hospitalizations while improving patients' quality of life and reducing HF-associated healthcare costs (17, 18).

The multidisciplinary HF clinic GEstIC (*Grupo de Estudo da Insuficiência Cardíaca*, meaning Heart Failure Study Group) was implemented at *Centro Hospitalar Universitário de Santo António* in 2013 and is composed of several healthcare professionals, including physicians from different specialties (internal medicine, cardiology, nephrology, psychiatry, physical medicine and rehabilitation and palliative care), nurses, a physiotherapist, a social worker, and a nutritionist. Most patients admitted to GEstIC proceed from the Internal Medicine Department after HF hospitalization and are managed according to a structured program, as per the guidelines of the cardiology societies for multidisciplinary clinics and HF management (2, 3, 16, 19). The GEstIC organization and HF management program was published previously (16). Briefly, like similar HF clinics, GEstIC has a comprehensive and multidisciplinary approach consisting of structured follow-up, patient education, therapy optimization, psychological support, and efficient access to healthcare. Patients are identified through electronic medical records after hospitalization for HF and, upon discharge, a GEstIC nurse initiates contact, providing education and a direct line to the team. The program encourages self-management and healthy habits and instruct patients and caregivers to notify the team of worsening symptoms. Regular patient monitoring includes hospital visits and evaluation of laboratory values, patient-reported outcomes, and tests such as the 6-Minute Walking Test. Notably, GEstIC also collaborates with a cardiac rehabilitation unit, allowing eligible patients to access tailored rehabilitation programs. The main aim of this study was to characterize the impact of the GEstIC multidisciplinary clinic on the improvement of specific healthcare indicators, namely the number of all-cause hospitalizations, HF events (hospitalizations and urgent visits), as well as their associated costs, in a Portuguese public hospital. With this work, it is expected that policymakers and other healthcare organizations will recognize the benefits of this model of care and promote the implementation of similar solutions in other units. Ultimately, this will contribute to the improvement of the quality and sustainability of the national healthcare system.

## 2. Materials and methods

### 2.1. Study design and study population

This single-center, observational, retrospective study included all patients admitted to the GEstIC clinic at *Centro Hospitalar Universitário de Santo António*, a university hospital in Porto, between January 1, 2013, and February 28, 2019, with at least one year of follow-up. The COVID-19 pandemic period was excluded; in Portugal, first COVID-19 patient was diagnosed in March 2020. To be admitted to GEstIC, patients had to be 18 years of age or older and either be recently hospitalized for HF or have history of previous HF hospitalization or frequent urgent HF visits. Exclusion criteria included complete dependence on caregivers for activities of daily living and inability to communicate. The study protocol was approved by the local ethics committee and institutional review boards.

### 2.2. Study outcomes

The primary outcome of this study was to evaluate the impact of GEstIC on all-cause hospitalizations and HF events. The latter includes HF hospitalizations and urgent HF visits. An HF hospitalization was defined as an event in which the patient is admitted to the hospital for at least 24 h with a primary diagnosis of HF (new or worsening symptoms of HF on presentation and objective evidence of new or worsening HF by physical examination and/or laboratory criteria) and initiates or intensifies HF treatment. An urgent HF visit was defined as an emergency department visit with a primary diagnosis of HF, receiving intravenous diuretics, but does not meet the criteria for HF hospitalization.

The number of events and total days of HF hospitalization in the year before and the year after GEstIC admission were determined and compared. In addition, the impact of HF hospitalizations on the number of hospital beds released annually was estimated.

The impact of the GEstIC multidisciplinary clinic on the total cost of HF events was calculated based on the number of events and number of days spent in the hospital due to HF, considering €428.55 as the average daily cost of one patient hospitalized in the Internal Medicine Department and €239.64 the average cost of one visit to the Emergency Department of *Centro Hospitalar Universitário de Santo António* (average costs were provided by the Health Information Management Department of the hospital).

### 2.3. Data collection

The number of all-cause hospitalizations, HF hospitalizations, urgent HF visits, and days hospitalized due to HF, during the year before and the year after the first GEstIC visit, were retrospectively collected from the clinical records and hospital databases. Data collected at the time of GEstIC admission included age, sex, HF etiology, presence and type of comorbidities, New York Heart Association (NYHA) class, N-terminal-pro-B type natriuretic peptide (NT-proBNP) levels, estimated glomerular filtration rate (eGFR), and left ventricular ejection fraction (LVEF) values. HF diagnosis and classification followed the Universal definition and classification of HF consensus statement (18).

### 2.4. Statistical analysis

Continuous variables were summarized using mean, standard deviation (SD), median, and 25th and 75th percentiles (P25 and P75). Absolute and relative frequencies were used for categorical variables. The number of events before and after first GEstIC admission was compared using the nonparametric Wilcoxon signed rank test with continuity correction for paired samples.

The correlation between the variation in the number of HF hospitalizations and LVEF values at GEstIC admission was assessed using the nonparametric Wilcoxon rank-sum test with continuity correction (Mann–Whitney) for independent samples.

Total savings from HF-associated hospitalizations and urgent visits were determined based on the average cost of each event per day provided by the Health Information Management Department, which was in place in 2021.

A 5% significance level was used. Statistical analysis was performed with R® software version 4.2.3 (Vienna, Austria).

## 3. Results

### 3.1. HF patients demographic and clinical characterization at GEstIC admission

**Figure 1:**
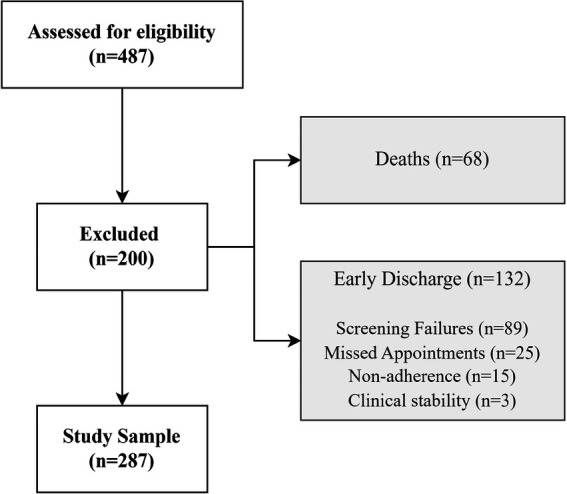
Flowchart of the study population.

A total of 487 patients were admitted to the GEstIC HF clinic between January 2013 and February 2019. Of these, 68 patients (14.0%) died within the first year and 132 (27.1%) did not complete a minimum of 365 days of follow-up. The remaining 287 patients (58.9%) met all inclusion criteria and constituted the study sample (Figure 1).

The patients' demographic and clinical characteristics at the time of admission to GEstIC are shown in Table 1. The median age of the study participants was 77 years, and the majority were female (56.8%). In terms of HF etiology, patients were primarily diagnosed with hypertensive (41.5%), ischemic (38.0%), and/or valvular (33.8%) heart disease, of which the latter were further identified as having aortic (64.9%) and/or mitral (54.3%) valvular disease. Most patients had NYHA functional class II symptoms (67.3%). According to the Universal definition and classification of Heart Failure and the 2021 ESC HF Guidelines classification, 48.4% of the patients had preserved ejection fraction (LVEF ≥50%) and 41.5% presented reduced LVEF (≤40%). Laboratory analyses revealed a median NT-proBNP level of 2,135 pg/mL. Hypertension (82.9%), iron deficiency (58.2%), and CKD (56.4%) were the most frequent comorbidities. Atrial flutter or fibrillation was present in 48.8% of patients.

**Table 1:** Patients’ demographic and clinical characteristics at GEstIC admission.

Characteristics	Value	*n* total
Age (years), median (P25; P75)	77 (68; 83)	287
Female, *n* (%)	163 (56.8)	287
Comorbidities		
Hypertension, *n* (%)	238 (82.9)	287
Iron deficiency, *n* (%)	96 (58.2)	165
CKD (eGFR <60 mL/min/1.73 m^2^), *n* (%)	162 (56.4)	287
eGFR 30–59	134 (46.7)	
eGFR 15–29	26 (9.1)	
eGFR <15	2 (0.7)	
Anemia, *n* (%)	148 (51.7)	286
Diabetes *mellitus*, *n* (%)	156 (54.9)	284
Atrial flutter/fibrillation, *n* (%)	140 (48.8)	287
COPD, *n* (%)	72 (25.1)	287
Cognitive decline, *n* (%)	27 (9.4)	287
HF etiology, *n* (%)		287
Hypertensive	119 (41.5)	
Ischemic	109 (38.0)	
Valvular	97 (33.8)	
Others	83 (28.9)	
NYHA functional class, *n* (%)		278
I	43 (15.5)	
II	187 (67.3)	
III	46 (16.5)	
IV	2 (0.7)	
LVEF, *n* (%)		287
≤40%	119 (41.5)	
41–49%	29 (10.1)	
≥50%	139 (48.4)	
NT-proBNP (pg/mL), median (P25; P75)	2,135 (789; 4,719)	261

CKD, chronic kidney disease; COPD, chronic obstructive pulmonary disease; eGFR, estimated Glomerular Filtration Rate; HF, heart failure; LVEF, left ventricle ejection fraction; NT-proBNP, N-terminal-pro-B type natriuretic peptide; NYHA, New York Heart Association.

### 3.2. Number of hospitalizations and urgent HF visits

**Figure 2:**
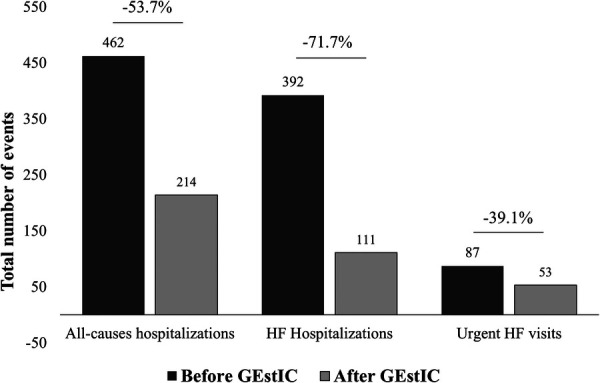
Number of events registered before and after GEstIC admission. Number of total all-cause hospitalizations, HF hospitalizations, and urgent HF visits registered for 287 HF patients one year before and one year after GEstIC first visit. *p*-values were determined using the Wilcoxon signed rank test with continuity correction.

In the year before GEstIC admission, a total of 462 all-causes hospitalizations, 392 HF hospitalizations, and 87 urgent HF visits were registered. At the end of the first year of follow-up in GEstIC, the number of such events significantly decreased to 214 (−53.7%), 111 (−71.7%), and 53 (−39.1%), respectively (Figure 2).

**Figure 3:**
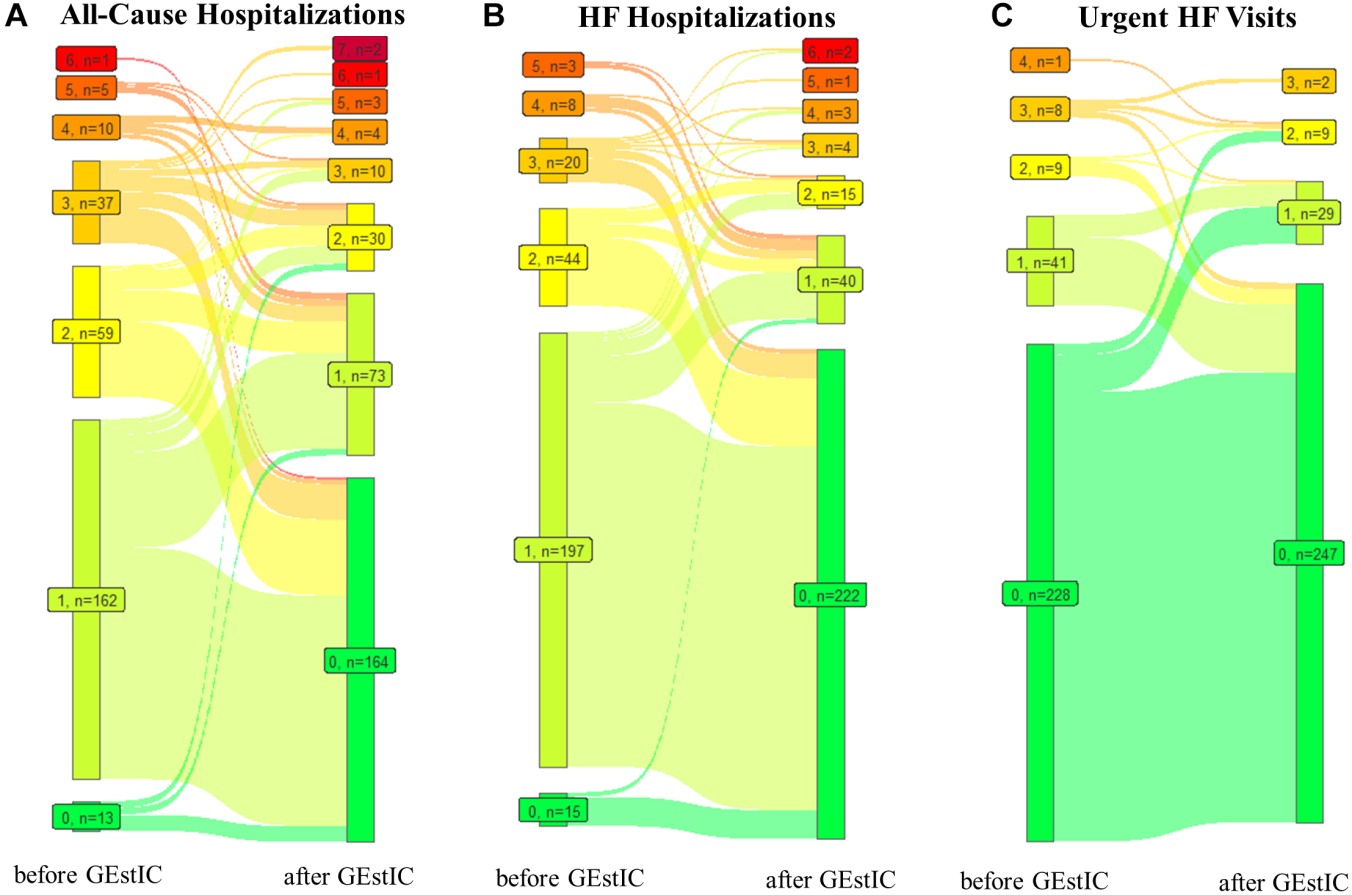
Number of patients with a certain number of all-cause hospitalizations, HF hospitalizations, and urgent HF visits registered before and after GEstIC admission. (**A**) Number of patients with zero to seven all-cause hospitalization events in the one year before GEstIC and one year after GEstIC admission. (**B**) Number of patients with zero to six HF hospitalization in the one year before GEstIC and one year after GEstIC admission. (**C**) Number of patients with zero to four urgent HF visits in the one year before GEstIC and one year after GEstIC admission. Data are presented as 0 events (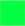), 1 event (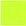), 2 events (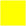), 3 events (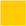), 4 events (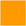), 5 events (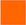), 6 events (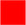), and 7 events (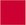).

For all-cause hospitalizations, the mean number of events per patient decreased significantly (*p* < 0.001) from 1.61 (SD ± 1.04) in the year before to 0.75 (SD ± 1.18) in the year after GEstIC admission (Table 2). Specifically, of the 287 patients, 195 decreased, 65 maintained, and 27 increased the number of hospitalizations. In addition, the number of patients who did not require all-cause hospitalizations increased from 13 (4.5%) to 164 (57.1%) (Figure 3A).

**Table 2:** Average number of all-causes hospitalizations and HF events per patient in the study population.

Events	Before GEstIC	After GEstIC	Variation	*p*-value*
All-causes hospitalizations, mean (SD)	1.61 (1.04)	0.75 (1.18)	0.86 (1.33)	<0.001
HF hospitalizations, mean (SD)	1.37 (0.88)	0.39 (0.91)	0.98 (1.07)	<0.001
Urgent HF visits, mean (SD)	0.30 (0.70)	0.18 (0.51)	0.12 (0.67)	0.005

* Wilcoxon signed rank test with continuity correction for paired samples.

The mean number of HF hospitalizations per patient decreased significantly (*p* < 0.001) from 1.37 (SD ± 0.88) before and 0.39 (SD ± 0.91) after GEstIC admission (Table 2). The total number of HF hospitalizations decreased in 229 patients, remained unchanged in 41 patients, and increased in 17 patients. The number of patients without HF hospitalizations between the two periods under comparison increased from 15 (5.2%) to 222 (77.4%) (Figure 3B).

Finally, the mean number of urgent HF visits per patient decreased significantly (*p* = 0.005) from 0.30 (SD ± 0.70) before to 0.18 (SD ± 0.51) after GEstIC admission (Table 2). Fourty-six patients reduced, 219 maintained, and 22 increased the number of urgent HF visits. The number of patients who did not require an urgent HF visit slightly increased from 228 (79.4%) to 247 (86.1%) (Figure 3C).

**Figure 4:**
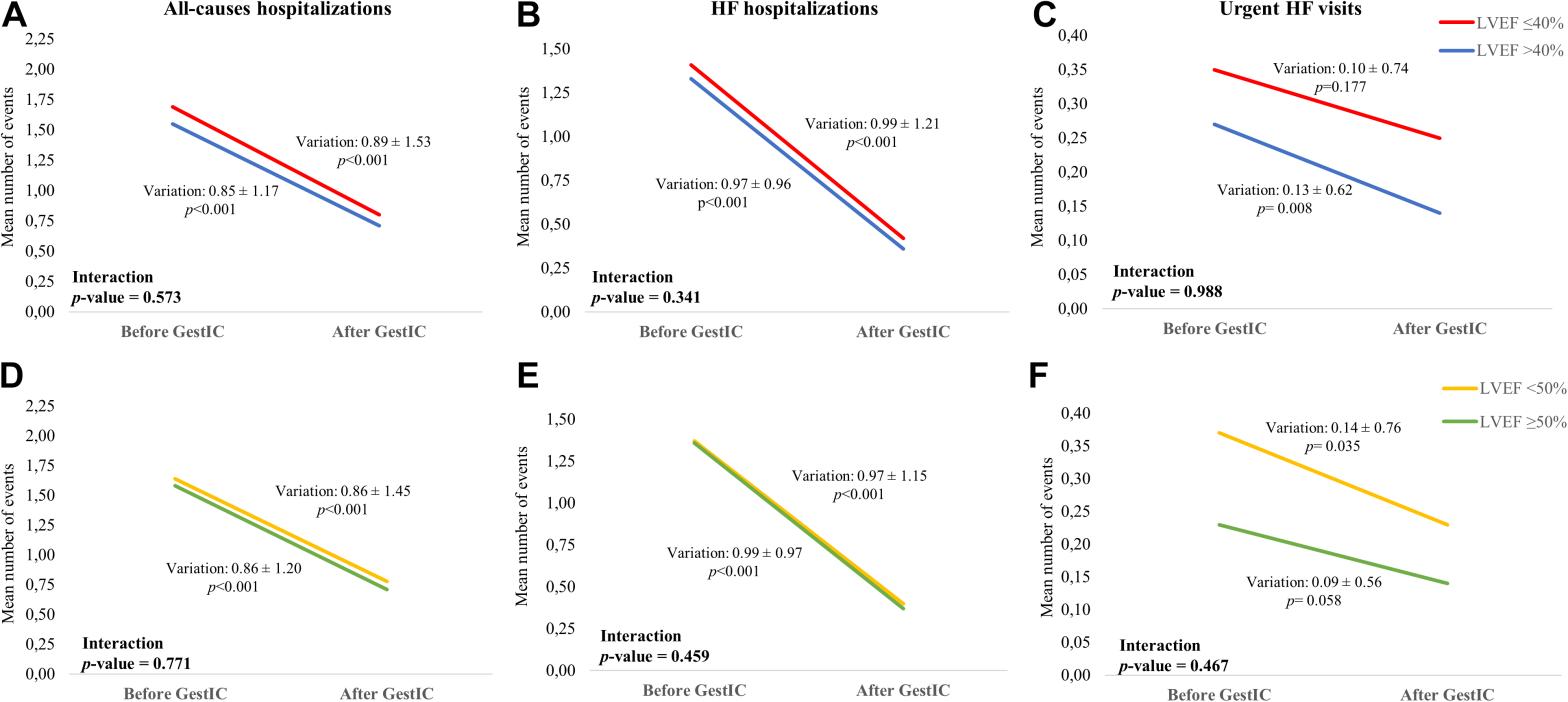
Mean number of all-causes hospitalizations and HF events per patient according to LVEF. (**A**) Mean number of all-cause hospitalizations, (**B**) HF hospitalizations, and (**C**) urgent HF visits during the one year before and one year after GEstIC admission in patients with LVEF ≤40% and >40%. (**D**) Mean number of all-cause hospitalizations, (**E**) HF hospitalizations, and **(F)** urgent HF visits during the one year before and one year after GEstIC admission in patients with LVEF <50% and ≥50%. *p*-values for the variation within each LVEF group were determined using the Wilcoxon signed rank test with continuity correction for paired samples; *p*-values for the comparison of the variation between LVEF groups were determined using the Wilcoxon rank-sum test with continuity correction (Mann-Whitney) for independent samples.

Next, we investigated the link between the number of all-cause hospitalizations, HF hospitalizations, and urgent HF visits per patient and the patient's LVEF at admission (Figure 4 and Supplementary Table S1). Results show that presenting a LVEF ≤40% or >40% did not affect the magnitude of the reduction in all-cause hospitalizations (*p* = 0.573, Figure 4A), HF hospitalizations (*p* = 0.341, Figure 4B), and urgent HF visits (*p* = 0.988, Figure 4C). Such observation was also valid when considering LVEF <50% and ≥50% (all-causes hospitalizations: *p* = 0.771, Figure 4D; HF hospitalizations: *p* = 0.459, Figure 4E; urgent HF visits: *p* = 0.467, Figure 4F). Regardless, within each group of patients, the mean number of registered events decreased in the year following GEstIC admission (Figure 4 and Supplementary Table S1), with the exception of the number of urgent HF visits in patients with LVEF ≤40% (*p* = 0.177, Figure 4C) and LVEF ≥50% (*p* = 0.058, Figure 4F), which was similar to the previous year.

### 3.3. Length of HF hospitalizations

The 392 HF hospitalizations registered in the year prior to GEstIC admission lasted for 4,488 days. Considering all days of HF hospitalizations, each patient spent on average (±SD) 15.6 ± 15.8 days hospitalized (Table 3). In contrast, during the first year of follow-up in GEstIC, the total days in hospital due to HF reduced to 864 days, corresponding to an 80.7% decrease. The mean (±SD) length of stay was reduced to 3.0 ± 7.8 days per patient, corresponding to a significant reduction of 12.6 days of hospital stays per patient (*p* < 0.001, Table 3). This reduction of 3,624 days of hospitalization due to HF translates into the release of 9.9 hospital beds in the year following admission to GEstIC. Of note, if we consider only the patients that had HF hospitalizations in the two periods (272 patients with HF hospitalizations before vs. 65 after GEstIC admission), the average length of stay decreased from 16.5 days to 13.3 days.

**Table 3:** Characterization of HF hospitalization length and cost of HF events of the study population before and after GEstIC admission.

Study population				
HF hospitalization	Before GEstIC	After GEstIC	Variation	*p*-value*
Total length, days	4,488	864	3,624	
Average length of stay/patient, days (SD)	15.6 (15.8)	3.0 (7.8)	12.6 (15.0)	<0.001
Total cost, €	1,923,332.40	370,267.20	1,553,065.20	
Average cost/patient, € (SD)	6,701.51 (6,770.01)	1,290.13 (3,331.21)	5,411.38 (6,423.61)	<0.001
Urgent HF visits				
Total cost, €	20,848.68	12,700.92	8,147.76	
Average cost/patient, € (SD)	72.64 (166.84)	44.25 (121.25)	28.39 (161.55)	0.005
HF events**^#^**				
Total cost, €	1,944,181.08	382,968.12	1,561,212.96	
Average cost/patient, € (SD)	6,774.15 (6,808.27)	1,334.38 (3,347.76)	5,439.77 (6,446.71)	<0.001

^#^ HF Events = HF hospitalizations + urgent HF visits.

* Wilcoxon signed rank test with continuity correction for paired samples.

### 3.4. Cost of HF events

After determining the impact of the GEstIC on the number and length of HF hospitalizations, the costs associated with these events were estimated. A total saving of €1,553,065.20 was observed based on an average daily cost of €428.55 per hospitalization and a reduction of 3,624 days of HF hospitalization days between the two study periods. A significant average cost reduction of €5,411.38 per patient in HF hospitalizations was achieved (*p* < 0.001, Table 3).

As for urgent HF visits, their average cost was estimated by the hospital to be €239.64 per visit. Thus, the reported reduction of 34 urgent HF visits translated into a saving of €8,147.76 in these events (*p* = 0.005, Table 3).

When the overhead costs are added together, the total saving associated with reducing HF events (hospitalizations and urgent visits) over the first year of follow-up of the 287 patients in the GEstIC was €1,561,212.96. This represents an average saving of €5,439.77 per patient (*p* < 0.001, Table 3).

## 4. Discussion

This study aimed to characterize the impact of the GEstIC multidisciplinary clinic on specific HF healthcare indicators and their associated costs. Here we present real-world evidence showing, for the first time in Portugal, that the multidisciplinary management of HF patients significantly reduces the number of all-cause hospitalizations and HF events (HF hospitalizations and urgent HF visits), the length of HF hospitalizations, and, consequently, the costs associated with such events.

The demographic and clinical analysis of the study sample showed that this was an elderly population (median age 77 years), predominantly female, with a high number of comorbidities. Around half of the patients had LVEF ≥50% and almost two-thirds had a mild limitation in their physical activity (NYHA II). This profile aligns with what has been reported as the European reality (20).

Substantial reductions in HF hospitalizations (−71.7%) and urgent HF visits (−39.1%) were observed after admission to GEstIC. Moreover, 77.4% of the patients did not require any hospitalization for HF during the one-year after admission to the clinic, compared to the 5.2% in the preceding period. Other reports have described similar findings, although not as significant as this. A systematic review examining the effect of multidisciplinary strategies on the outcomes of HF patients indicated a 26% reduction in HF hospitalizations under randomized clinical trial conditions (15). More satisfactory values were reported by McMurray et al*.* and Packer et al*.,* who observed a reduction in HF hospitalizations of around 30% when investigating the effect of new therapeutic options for HF (21–23). Data from a 3-month period of enrollment in a multidisciplinary community paramedicine program demonstrated a 25% reduction in the number of emergency department visits by patients with HF (24). In addition to the impact on the number of events, the length of stay for HF hospitalizations was also significantly shorter (−12.6 days of hospitalization/patient) during the first year of follow-up in GEstIC compared to the year before admission. This is particularly remarkable considering the patient demographics in a Portuguese internal medicine unit—predominantly elderly individuals with a variety of non-medical complexities (e.g., frailty, social isolation) and additional factors, such as hospital-acquired infections, that typically contribute to longer stays.

Considering these findings within their context, the main HF event in this population was hospitalization rather than urgent HF visits, which may be since most HF-related emergency visits ultimately lead to hospital admission. The implementation of multidisciplinary care had a positive impact mainly on reducing hospitalizations and, to a lesser extent, urgent HF visits. In addition, this multidisciplinary clinic did not significantly change the number of urgent HF visits in patients with LVEF ≤40% and ≥50%. Notably, during the study period, there was no alternative to the emergency department for the evaluation of HF decompensation events, as the GEstIC work was based in an outpatient ambulatory setting. Altogether, it is reasonable to speculate that HF events after GEstIC admission were probably less severe than those occurring in the previous year, leading to a lower number of hospitalizations. This idea is further supported by the significantly shorter length of stay for HF hospitalization after GEstIC admission.

The multidisciplinary management of these patients also led to a 53.7% reduction in all-cause hospitalizations. In the first year after GEstIC admission, 57.1% of patients did not require any hospitalization, opposed to 4.5% in the previous year. McAlister et al. observed a similar trend and reported a 19% reduction in all-cause hospitalizations upon implementation of multidisciplinary management for HF patients (15). In fact, multidisciplinary follow-up of HF patients by a cardiologist and a geriatrician significantly reduced all-cause hospitalizations compared with follow-up by a cardiologist only, as demonstrated by Herrero-Torrus et al. (25).

In this study, the multidisciplinary management of HF patients was beneficial for all patients, including those with preserved LVEF, who reported fewer all-cause hospitalizations and HF events one-year after admission to GEstIC. Until the publication of the results from the EMPEROR-Preserved study in 2021, no drug treatment had been shown to significantly improve the prognosis of HF patients with LVEF >40% (26). Nevertheless, data from the present study, which reports up to February 2020 and before the knowledge that empagliflozin and dapagliflozin can reduce mortality and HF events in patients with HF and LVEF >40%, showed that admission to GEstIC reduced the number of all-cause hospitalizations and HF events in this group of patients in the first year of follow-up. These findings emphasize the importance of an all-encompassing, patient-centered, and multidisciplinary approach to HF patient management. Our results indicate that the implementation of such a program contributes significantly to the reduction in hospitalizations and HF-related events, thereby contributing to an overall improvement in patients' health status and quality of life.

One of the most significant outcomes of reducing hospitalizations and urgent HF visits is the financial impact on the public healthcare system. In 2014, the direct cost of HF to the Portuguese public healthcare system was €299 million. Here we show that the reduction of HF-related events (hospitalizations and urgent visits) after the admission of 287 patients to the GEstIC during the first year of follow-up represented an economic saving of €1,561,212.96 in a single hospital. Of note, if the costs of all-cause hospitalizations had been included in this estimate, the total savings would have been substantially higher. Other studies have reported a reduction in costs after implementing multidisciplinary clinics. Weinstein et al. demonstrated a 27% decrease in hospitalization costs after a one-year follow-up of a community-based HF multidisciplinary clinic (27). Ledwidge et al*.* calculated an overall cost/saving of €586 per hospitalization prevented and €729 per patient treated during a 3-month period of multidisciplinary care of HF patients (28). Despite variations in study implementation and design, daily hospitalization costs, and multidisciplinary clinic costs, the consensus is that replication of GEstIC in other Portuguese public institutions would reduce the economic burden on the public health system, saving millions of euros, while reducing the use of healthcare resources, being human or logistical.

In addition to the beneficial effects of physical exercise, regular assessment of health and quality of life, and adherence to treatment, among other factors, in reducing mortality and improving patients' well-being and quality of life, multidisciplinary clinics also have an important role in achieving these results, similar to angiotensin-converting enzyme inhibitors. (14, 15, 19). In Portugal, although not extensively described, multidisciplinary clinics for HF patients were shown to reduce the risk of mortality and short-term hospitalization (16).

Following the introduction of GEstIC, 487 individuals were admitted within the initial six-year period, corresponding to this study period. Notably, during the first year of follow-up, the mortality rate was recorded at 14%, significantly lower than the 34.4% that we reported in an unselected HF population admitted to our Internal Medicine Department in the year before GEstIC implementation and the 22.8% reported in a recent real world study of HF patients from Sweden (29, 30).

Further analysis would be important to better understand the full potential of this HF multidisciplinary clinic, particularly on clinical and patient-reported outcomes. In addition, the long-term follow-up benefits in terms of HF complications and comorbidities, patients’ overall survival, and human and economic resources use should be evaluated. Finally, a continuous re-evaluation of the results of this study would be desirable to include more patients in this analysis and thus contribute to more robust conclusions.

The major strength of the study lies in its design, which involves comparing the same group of patients before and after their admission to GEstIC. This approach minimizes the potential for bias. In addition, the researchers used real-world data collected from medical records. However, there are several limitations to acknowledge. The study did not provide a comprehensive characterization of additional costs associated with patients' treatments, interventions, or follow-up visits within the GEstIC program, including cardiovascular medications, iron supplementation, or cardiac rehabilitation, which could have influenced the observed positive outcomes. While our study aimed to assess the direct impact on hard clinical events, future cost-benefit analyses should consider incorporating these elements to provide a more holistic understanding of the program's economic implications. Secondly, this study did not evaluate the frequency of patients with LVEF ≤40% receiving prognostic modifying drugs or patients with LVEF >40% undergoing empagliflozin or dapagliflozin treatment for diabetes. Despite these limitations, it is important to note that the primary objective of the HF multidisciplinary clinic is to optimize treatment, ensuring that patients receive the best possible care. Lastly, the cost estimate considered the average cost of treating any patient, with any disease, in the Internal Medicine Department and the Emergency Department, which may be higher or lower than treating a HF patient, *per se*.

Future research is important to explore the combined effects of specialized multidisciplinary care and improved organizational aspects in HF clinics like GEstIC. This exploration will allow for a better understanding of their respective influences on patient outcomes, in addition to characterizing patient pathways and evaluating potential enhancements in health-related quality of life, exercise capacity, and mental health.

In conclusion, multidisciplinary clinics have shown great benefits in the diagnosis, treatment, and follow-up of HF patients. In addition to improving clinical outcomes by reducing the number of events (hospitalizations and urgent visits), these clinics also have the added value of helping healthcare institutions and other stakeholders better manage human, logistical, or financial resources. Therefore, replicating this model in other hospitals would certainly contribute to the sustainability of health systems.

## Data Availability

The raw data supporting the conclusions of this article will be made available by the authors, without undue reservation.
