# Supplementary material for: Impact of a heart failure multidisciplinary clinic on the reduction of healthcare-related events and costs: the GEstIC study
Source: Front Cardiovasc Med. 2023 Sep 29;10:1232291. doi: 10.3389/fcvm.2023.1232291 (PMC10576556; doi:10.3389/fcvm.2023.1232291)
Supplement: Supplementary file 1 [file Table1.doc]

Supplementary Material

Impact of a heart failure multidisciplinary clinic on the reduction of healthcare-related events and costs: the GEstIC study

Rita Rego^1^, Nuno Pereira^1^, António Pinto^2^, Sofia Pereira^3^, Irene Marques^1,2,4*^

^1^Serviço de Medicina Interna, Centro Hospitalar Universitário de Santo António, Porto, Portugal;

^2^Unidade Multidisciplinar de Investigação Biomédica – Instituto de Ciências Biomédicas de Abel Salazar (ICBAS), Universidade do Porto, Porto, Portugal

^3^Serviço de Medicina Interna, Centro Hospitalar de Tondela Viseu, Viseu, Portugal

^4^ ITR-Laboratory for Integrative and Translational Research in Population Health, Porto, Portugal

**Supplementary Table 1. Mean number of all-causes hospitalizations and HF events per patient according to LVEF.**

| LVEF ≤40% Population | | | | |
| --- | --- | --- | --- | --- |
| **Events** | **Before GEstIC** | **After GEstIC** | **Variation** | **p-value*** |
| All-causes hospitalizations, mean (SD) | 1.69 (1.11) | 0.80 (1.38) | 0.89 (1.53) | <0.001 |
| HF hospitalizations, mean (SD) | 1.41 (0.90) | 0.42 (1.12) | 0.99 (1.21) | <0.001 |
| Urgent HF visits, mean (SD) | 0.35 (0.74) | 0.25 (0.61) | 0.10 (0.74) | 0.177 |
| **LVEF >40% Population** | | | | |
| **Events** | **Before GEstIC** | **After GEstIC** | **Variation** | **p-value*** |
| All-causes hospitalizations, mean (SD) | 1.55 (0.99) | 0.71 (1.01) | 0.85 (1.17) | <0.001 |
| HF hospitalizations, mean (SD) | 1.33 (0.87) | 0.36 (0.74) | 0.97 (0.96) | <0.001 |
| Urgent HF visits, mean (SD) | 0.27 (0.66) | 0.14 (0.41) | 0.13 (0.62) | 0.008 |
| **LVEF <50% Population** | | | | |
| **Events** | **Before GEstIC** | **After GEstIC** | **Variation** | **p-value*** |
| All-causes hospitalizations, mean (SD) | 1.64 (1.03) | 0.78 (1.29) | 0.86 (1.45) | <0.001 |
| HF hospitalizations, mean (SD) | 1.37 (0.84) | 0.40 (1.05) | 0.97 (1.15) | <0.001 |
| Urgent HF visits, mean (SD) | 0.37 (0.79) | 0.23 (0.58) | 0.14 (0.76) | 0.035 |
| **LVEF ≥50% Population** | | | | |
| **Events** | **Before GEstIC** | **After GEstIC** | **Variation** | **p-value*** |
| All-causes hospitalizations, mean (SD) | 1.58 (1.05) | 0.71 (1.04) | 0.86 (1.20) | <0.001 |
| HF hospitalizations, mean (SD) | 1.36 (0.92) | 0.37 (0.74) | 0.99 (0.97) | <0.001 |
| Urgent HF visits, mean (SD) | 0.23 (0.57) | 0.14 (0.40) | 0.09 (0.56) | 0.058 |

*Wilcoxon signed rank test with continuity correction for paired samples
